# Precursor T-Cell Lymphoblastic Lymphoma Presenting as Cardiac Tamponade in a 25-Year-Old Male: A Case Report and Review of Literature

**DOI:** 10.14740/wjon785w

**Published:** 2014-06-25

**Authors:** Sakshi Kapur, Miles B. Levin

**Affiliations:** a Department of Internal Medicine, Overlook Medical Center, 99 Beauvoir Ave, Summit, New Jersey 07902, USA; b Division of Pathology, Overlook Medical Center, 99 Beauvoir Ave, Summit, New Jersey 07902, USA

**Keywords:** Precursor T-cell lymphoblastic lymphoma, Pleural and pericardial effusion, Cardiac tamponade, Pericardial window

## Abstract

Precursor T-cell lymphoblastic lymphoma (LBL) and T-cell acute lymphoblastic leukemia (ALL) are considered same disease with different clinical presentations. Clinically, a case is defined as lymphoma if there is a mass lesion in the mediastinum or elsewhere and < 25% blasts in the bone marrow. Whereas, bone marrow with > 25% blasts with or without mediastinal masses is classified as T-cell ALL. Mediastinal masses caused by T-cell LBL can lead to complications such as superior vena cava syndrome, tracheal obstruction, pericardial effusion and tamponade. We report an unusual case of a 25-year-old male with no significant past medical history, who presented with clinical features of cardiac tamponade. Work-up revealed a massive pericardial effusion and a mass arising in the anterosuperior mediastinum. Patient underwent an emergent subxiphoid pericardial window and approximately 1 L of hemopericardium was drained. Histopathology (pericardial tissue) and flow cytometry on the pericardial fluid were compatible with a precursor T-cell LBL. Although pericardial involvement by lymphoma/leukemia is a very rare complication, cases have been reported with both lymphoma and acute/chronic leukemia. Our paper highlights cardiac tamponade as one of the life-threatening complications associated with a precursor T-cell LBL.

## Introduction

Up to 70% of patients with precursor T-cell lymphoblastic lymphoma (LBL) develop masses in the medistinum. Usually these masses are anterior, bulky and are associated with massive pleural and pericardial effusions. Mediastinal masses caused by precursor T-cell LBL can lead to complications such as superior vena cava syndrome, tracheal obstruction, pleural/pericardial effusion and tamponade. Although bone marrow is not involved initially, 60% of the patients eventually develop bone marrow infiltration during the course of disease. Combination chemotherapy produces an excellent response in these patients, but relapse is common.

## Case Report

A 25-year-old male with no significant past medical history presented to our hospital with progressively worsening shortness of breath over 3 months. Patient also complained of associated coughing paroxysms over the last 2 months. However, he denied any fever, chills, recent travel or weight loss.

Physical examination revealed an average sized male with difficulty breathing. Vitals on presentation were as follows: temperature: 97.6 F, blood pressure: 96/58 mm Hg and positive pulsus paradoxus, pulse: 120 beats per minute and respiratory rate: 28 per minute. Oxygen saturation was 92% on room air.

Systemic examination: head and neck exam was positive for jugular venous distention; on auscultation of lungs, there were decreased breath sounds bilaterally, and both lung fields were dull on percussion (left >> right). Cardiac exam revealed muffled heart sounds and a faint pericardial friction rub. Abdomen was soft, non-tender with a positive hepato-jugular reflex. Extremities revealed no clubbing, cyanosis or pedal edema.

**Figure 1:**
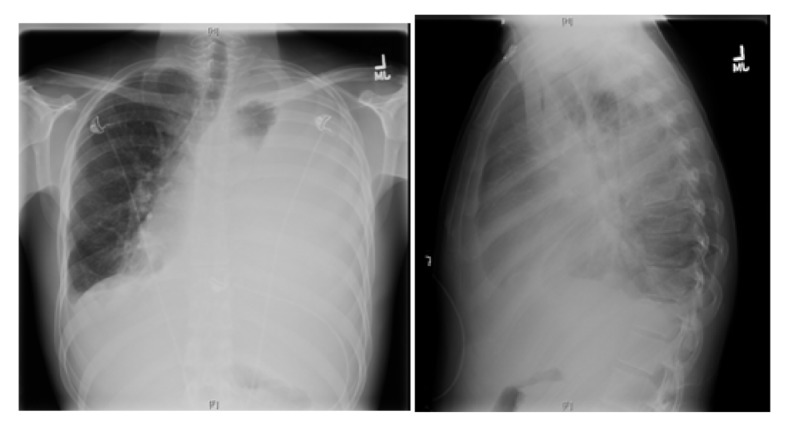
X-ray chest showing near complete opacification of left lung due to massive pleural effusion with shift of mediastinum to right.

Further work-up revealed a normal complete blood count and comprehensive metabolic panel. X-ray chest showed almost complete opacification of the left lung due to large pleural effusion, and shift of mediastinum toward right due to the effusion (Fig. 1).

**Figure 2:**
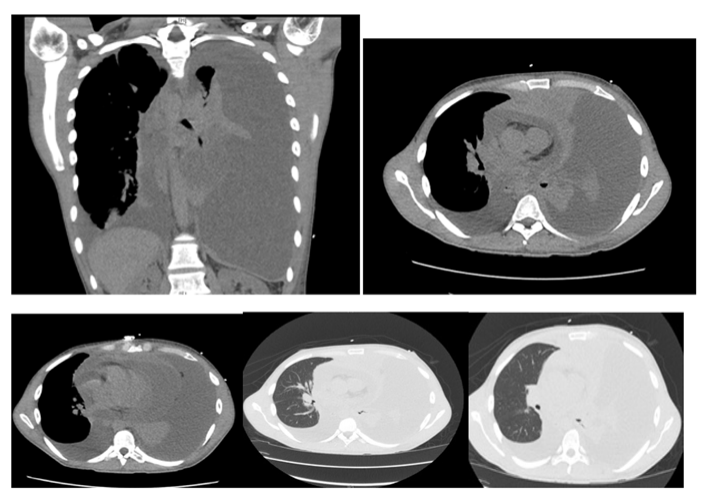
Computer tomography of the chest showing massive left pleural effusion, moderate right pleural effusion, massive pericardial effusion and a mass arising in the anterosuperior mediastinum.

Computer tomography of the chest revealed: massive left pleural effusion with left lung atelectasis and shift of mediastinum from left to right, moderate right pleural effusion, massive pericardial effusion and a mass arising in the anterior mediastinum (Fig. 2).

**Figure 3:**
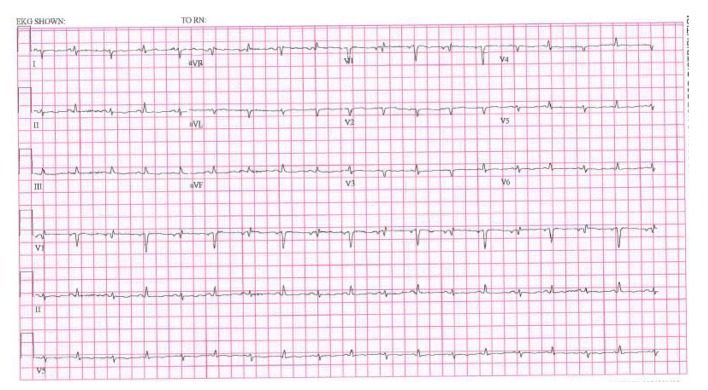
EKG showing low voltage, electrical alternans and sinus tachycardia.

EKG showed sinus tachycardia, low voltage and electrical alternans (Fig. 3).

**Figure 4:**
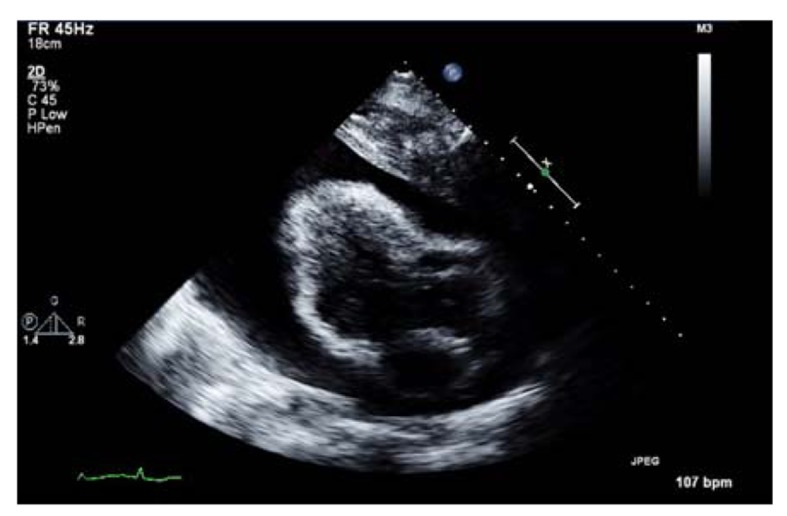
Transthoracic echocardiogram showing massive pericardial effusion.

Transthoracic echocardiogram results confirmed a massive pericardial effusion measuring 5.5 - 6 cm (in largest diameter from apex) and a swinging heart (Fig. 4). These findings were compatible with a cardiac tamponade secondary to massive pericardial effusion.

**Figure 5:**
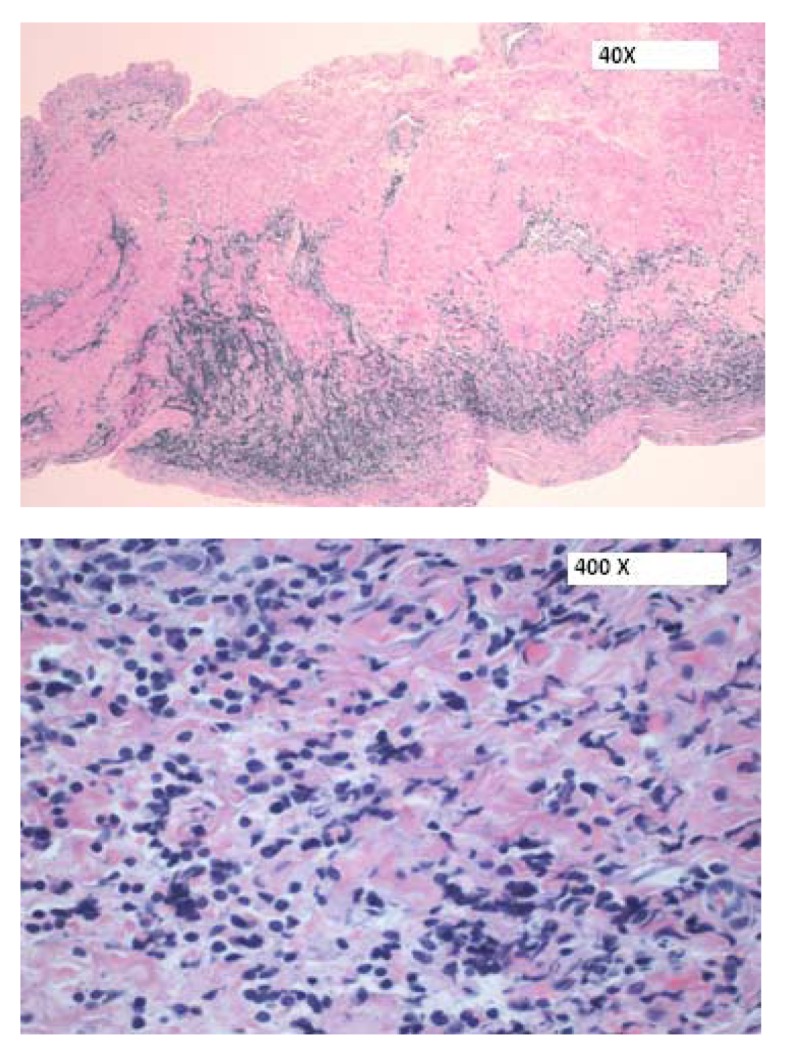
H&E sections of the pericardium showing a focal infiltrate of medium-sized cells within the pericardial wall.

**Figure 6:**
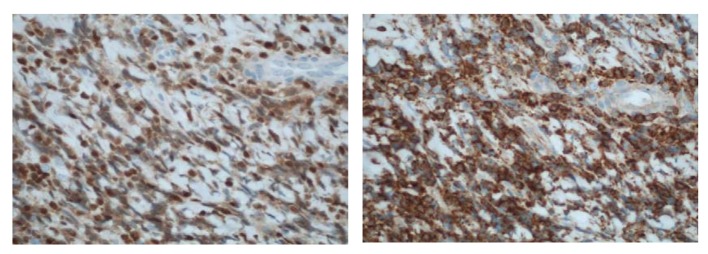
(× 400) Immunohistochemical stains showing (a) an immature CD99+, (b) TdT+ population consistent with a T-cell LBL.

Patient underwent an emergent subxiphoid pericardial window and approximately 1 L of hemopericardium was drained. Bilateral chest tubes were also inserted for pleural effusion. Following drainage of his effusions (pericardial and pleural), patient’s condition improved significantly. Histopathology (pericardial tissue) results were compatible with a precursor T-cell LBL (Fig. 5, 6).

**Figure 7:**
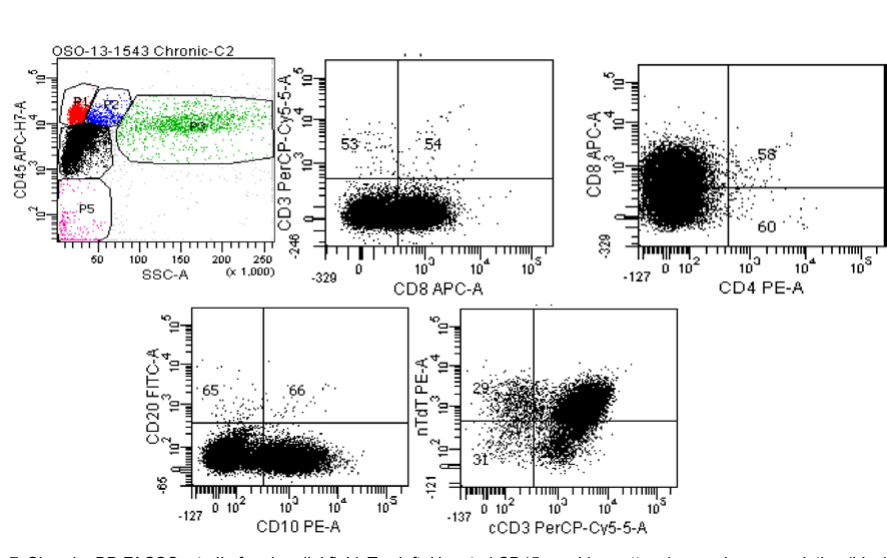
Six color BD FACSCanto II of pericardial fluid. Top left: Ungated CD45 vs. side scatter shows a large population (black dots) in the dim CD45/low side scatter (blast) gate. Top middle: Gating only this dim CD45 population show the cells are negative for surface CD3, positive for CD8, negative for CD4 (top right) and positive for CD10 (lower left). The lower right scattergram shows that although the population of interest was negative for surface CD3, cytoplasmic CD3 is present along with nuclear *terminal deoxynucleotidyl transferase* (nTdT).

Flow cytometry on the pericardial fluid also confirmed findings compatible with a precursor T-cell LBL (Fig. 7).

An oncology consultation was requested. Both bone marrow biopsy and lumbar puncture were negative for lymphoma. Bone scan revealed no evidence of metastasis. Serum LDH and uric acid were 210 U/L (100 - 190 U/L) and 4.4 mg/dL (3.5 - 8.5 mg/dL), respectively. Patient was treated with combination chemotherapy comprising of vincristine, asparaginase, doxorubicin and prednisone. He also received intrathecal methotrexate.

## Discussion

Precursor T-cell LBL and T-cell acute lymphoblastic leukemia (ALL) are considered same disease with different clinical presentations [1]. Clinically, a case is defined as lymphoma if there is a mass lesion in the mediastinum or elsewhere and < 25% blasts in the bone marrow. Whereas, bone marrow with > 25% blasts with or without mediastinal masses is classified as T-cell ALL. There is significant biological and clinical overlap between precursor T-cell LBL and T-cell ALL. Although some patients present with predominantly lymphomatous involvement (mediastinal mass or another defined lesion), most have or later develop marrow involvement. Similarly, patients who present with leukemia may have or develop extramedullary tumors.

Precursor T-cell LBL occurs most frequently in late childhood, adolescence or young adulthood. The male to female ratio is 2:1. Precursor T-cell LBL is considered a type of non-Hodgkin’s lymphoma, and constitutes 2% of these tumors [2]. The incidence in United States is reported as three cases per million persons per year, and does not vary by ethnicity [3].

**Figure 8:**
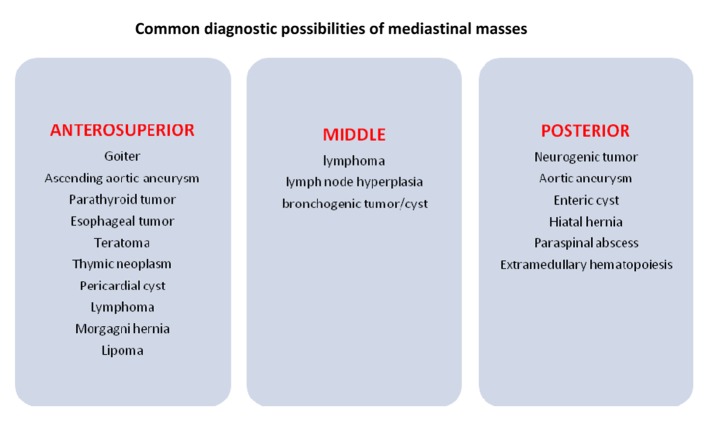
Differential diagnosis for a mediastinal mass.

Patients usually present with lymphadenopathy in cervical, supraclavicular and axillary regions [4]. Up to 70% of patients develop a mediastinal mass [4]. In most patients, the mediastinal mass is anterior, bulky, and is associated with pleural effusions. It is important to distinguish a mediastinal mass caused by precursor T-cell LBL from other causes, as T-cell LBL warrants aggressive therapy (Fig. 8).

Mediastinal masses caused by precursor T-cell LBL can lead to complications such as superior vena cava syndrome, tracheal obstruction, pericardial effusion and tamponade. Erdogan et al reported an unusual case of a 20-year-old male, who presented with cardiac tamponade secondary to chylous pericardial effusion. Flow cytometry results on pericardial fluid were compatible with a precursor T-cell LBL [5]. Sogut et al reported another case of a 3-year-old girl who presented with cardiac tamponade secondary to pericardial effusion. Clinical evaluation and laboratory results revealed T-cell ALL with pericardial invasion [6]. Mancuso et al reported yet another case of cardiac tamponade secondary to pericardial effusion in a patient with precursor T-cell ALL [7]. However, pericardial involvement remains a rare manifestation of lymphomas and leukemias. Cassis et al reported a rare case of massive hemopericardium in a patient with chronic myelogenous leukemia [8]. In a large autopsy study of 420 patients with acute leukemia, Roberts et al reported leukemic infiltration in hearts of 37% (156 patients) of patients [9]. In another study by Chu et al, 17 patients with pericarditis and cardiac tamponade were evaluated. These included nine patients with ALL, five with acute myeloid leukemia, two with chronic myelogenous leukemia and one patient had chronic lymphogenous leukemia [10].

Most patients (80%) with precursor T-cell LBL present with stage III or IV disease, and almost 50% develop type B symptoms. Although bone marrow is usually uninvolved at the time of diagnosis, approximately 60% of the patients eventually develop bone marrow infiltration and subsequent leukemic phase indistinguishable from T-cell ALL [11]. Evaluation of spinal fluid is essential to rule out CNS involvement, especially in patients with bone marrow involvement, as the incidence of CNS infiltration is high in these patients.

On histochemistry, the blasts in precursor T-cell LBL often show positivity on periodic acid Schiff (PAS) staining, variable positivity for non-specific esterase and Sudan black B, and negativity for myeloperoxidase. On flow cytometry, the lymphoblasts are typically positive for CD7 and surface or cytoplasmic CD3, and variably express CD2, CD5, CD1a, CD4 and/or CD8. A small subset of these tumors also express CALLA (CD10). Rarely, precursor T-cell LBL can express antigens present on NK cells such as CD16 and CD 57.

Various prognostic factors have been studied for precursor T-cell LBL. In UKALL XII/ECOG 2,293 trial, 356 patients were evaluated for their prognostic features. Survival was significantly shorter for females and patients > 35 years of age, positivity of blasts for CD1a and lack of expression of CD13 were associated with a significantly better survival and patients with a matched sibling donor had a better 5-year survival rate [12].

Precursor T-cell LBL warrants aggressive management. Combination chemotherapy produces an excellent response in these patients, but relapse is common [13, 14]. In children with precursor T-cell LBL, chemotherapy regimens used for treatment of T-cell ALL have produced 5-year disease-free survival rates ranging from 60% to 80%. T-cell ALL regimens may be equally effective in adults, although many adults are treated with regimens traditionally designed for diffuse intermediate-grade lymphoma such as cyclophosphamide, doxorubin, vincristine and prednisone (CHOP) or CHOP-like regimens. Depending on the regimen, response rate in adults ranges from 55% to 95%, with leukemia-type regimens producing rates greater than 70%. Hyper-CVAD (fractionated cyclophosphamide vincristine, adriamycin and dexamethasone) has also been used in patients with precursor T-cell LBL. In a study by Thomas et al, hyper-CVAD regimen generated a 100% initial response with a 70% overall survival benefit, but 40-60% of patients eventually relapsed [15]. Complications encountered during the treatment of precursor T-cell LBL include neutropenic fever, opportunistic infections, bleeding and tumor lysis syndrome.

### Conclusion

Precursor T-cell LBL can produce life-threatening complications such as cardiac tamponade. Although cases have been reported in literature, pericardial effusion secondary to precursor T- cell LBL causing cardiac tamponade remains a rare presentation. Our patient not only developed cardiac tamponade secondary to massive pericardial effusion but also had evidence of direct pericardial infiltration by the lymphoma, as noted on pericardial biopsy. Our paper once again highlights cardiac tamponade as one of the life-threatening complications associated with a precursor T-cell lymphoblastic lymphoma.
